# Refractory Thyroid Storm Due to Graves Disease in a Patient With Thymic Lymphoepithelial Carcinoma

**DOI:** 10.1210/jcemcr/luad053

**Published:** 2023-08-02

**Authors:** Kenda Alkwatli, Misbah Azmath, Prashant Grover, Pooja Luthra

**Affiliations:** Cleveland Clinic Foundation, Division of Endocrinology, Diabetes, and Metabolism, Cleveland, OH 44106, USA; Division of Endocrinology, Diabetes and Metabolism, University of Connecticut School of Medicine, Farmington, CT 06032, USA; Trinity Health of New England Medical Group, Division of Pulmonary and Critical care, Hartford, CT 06105, USA; Division of Endocrinology, Diabetes and Metabolism, University of Connecticut School of Medicine, Farmington, CT 06032, USA

**Keywords:** thymic lymphoepithelial carcinoma, thyroid storm, Graves disease, plasma exchange

## Abstract

Thyroid storm is a life-threatening endocrine emergency that warrants early clinical recognition and aggressive intervention. We present a 64-year-old female with no known history of thyroid disease, who presented to her primary care physician with dyspnea on exertion and was found to have an anterior mediastinal mass. She had elective thymectomy. Pathology confirmed thymic lymphoepithelial carcinoma. Postoperatively, she developed altered mental status, fever, and atrial fibrillation with marked elevation of thyroid hormones, consistent with thyroid storm. She decompensated rapidly and was treated aggressively with standard therapies for thyroid storm, including beta-blockers, methimazole, cholestyramine, steroids, and iodine, with poor response. The patient eventually underwent 4 sessions of therapeutic plasma exchange (TPE) with marked improvement in her symptoms. This case reports a possible association between thymic lymphoepithelial carcinoma and Graves disease and highlights the utility of TPE in cases of severe thyroid storm that are refractory to traditional treatments. We learn from this case that evaluating thyroid function tests in patients with thymic or mediastinal masses before surgery might be helpful. TPE should be considered in patients with thyroid storm refractory to traditional therapies.

## Introduction

Thymic lymphoepithelial carcinoma (TLEC) is a rare type of thymic carcinoma [1]. An association between thymic hyperplasia and Graves disease has been well described in the literature [2]. However, the coexistence of thymic cancer with Graves disease has not been previously reported. Thyroid storm is a rare and life-threatening endocrine emergency. The myriad clinical manifestations and rarity of severe thyroid storm make the diagnosis and effective treatment challenging. Untreated, mortality with thyroid storm can be as high as 8% to 30% [3]. Management of thyroid storm usually warrants admission to the intensive care unit and the use of anti-thyroid drugs in addition to beta-adrenergic blockers, Lugol's iodine, and intravenous steroids [4]. Therapeutic plasma exchange (TPE) is rarely indicated in the treatment of refractory thyroid storm and has often been used as a bridge to thyroidectomy.

We report a patient with TLEC who developed severe thyroid storm in the setting of undiagnosed Graves disease and was treated successfully with TPE and total thyroidectomy.

## Case Presentation

A 64-year-old female presented with 2 months of worsening dyspnea on exertion. Seven weeks prior to presentation, she had an initial coronary computed tomography angiogram, which showed a right paratracheal mass. Four weeks prior to presentation, she had a contrast-enhanced computed tomography (CT) of the chest, which showed a 4-cm lobulated, irregular right paratracheal mass with compression of the trachea and the right brachiocephalic vein. She was admitted to the hospital for elective thymectomy via a sternotomy. Pathology confirmed complete excision of lymphoepithelial-like thymic cancer. One day after surgery, the patient developed rapid atrial fibrillation. Laboratory evaluation showed low thyrotropin (TSH) of 0.05 uIU/mL (reference range, 0.35-5.50), elevated total thyroxine (T4) of 24.9 ug/dL [320.51 nmol/L] (reference range, 4.5-10.9 g/dL; 57.9-140.3 nmol/L), and total triiodothyronine (T3) 261 ng/dL [400.93 nmol/L] (reference range, 40-181 ng/dL: 61.4-278 nmol/L). Thyroid stimulating immunoglobulin was elevated at 6.75 IU/L (< 0.10 IU/L). The patient did not have a prior history of thyroid dysfunction. However, she was noted to have resting tachycardia on admission. She was diagnosed with Graves disease with significant thyrotoxicosis. She was started on methimazole 15 mg twice daily, and the dose was titrated up to 20 mg 3 times a day. On day 3 post surgery, she developed shortness of breath with hypoxia (SpO_2_ 85%) and mental status changes. The Burch and Wartofsky point scale was 55, suggestive of a thyroid storm. She was started on intravenous hydrocortisone 100 mg every 8 hours and oral cholestyramine 4 gm 4 times daily. Liver function tests were within normal limits. However, her mental status continued to worsen with increasing lethargy, fever of 101 °F, atrial fibrillation with a rapid ventricular response, and hypoxia requiring emergent intubation. Repeat Burch and Wartofsky point scale was 95 without other new precipitating factors or other causes for her worsening clinical status. On day 9 post surgery, total T4 was still significantly elevated at 17.2 mcg/dL (227.8344 nmol/L). Lugol's iodine solution was added to the treatment regimen. However, despite aggressive treatment for thyroid storm, she continued to spike high-grade fever with no improvement in mental status and no significant changes in total and free T4 levels.

## Treatment

She was then started on therapeutic plasma exchange (TPE) on Day 12 and had 4 sessions.

## Outcome and Follow-up

**Figure 1.**
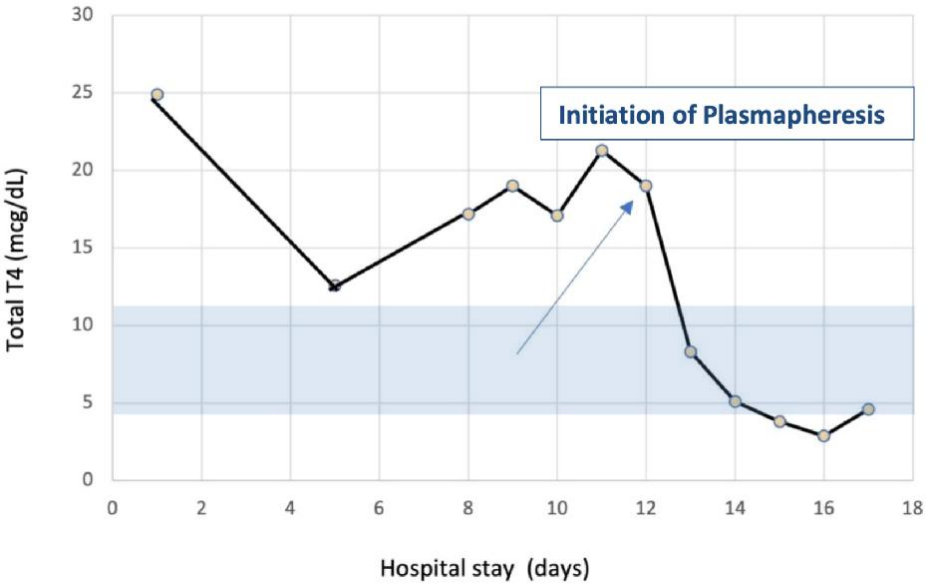
Total T4 measurements throughout the patient's hospital stay.

**Figure 2.**
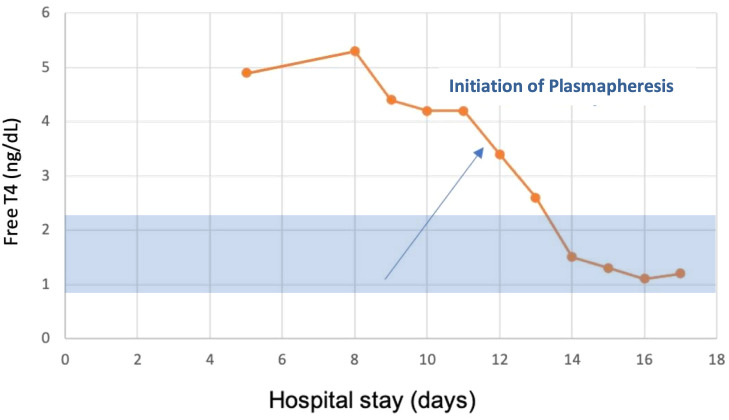
Free T4 measurements throughout the patient's hospital stay.

**Figure 3.**
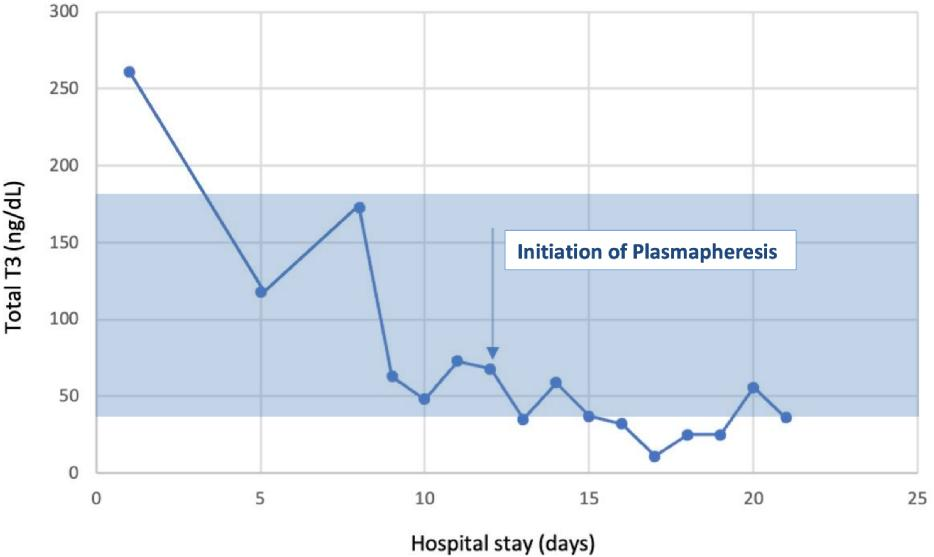
Total T3 measurements throughout the patient's hospital stay.

After the first TPE session, her total T4 level decreased by 60%, and by the fourth treatment, her total T4 normalized at 4.6 ug/dL [59.211 nmol/L], and total T3 decreased to 35 ng/dL [53.764 nmol/L] (Fig. 1-3). Her mental status and hemodynamic status markedly improved, and she was extubated. She eventually underwent a total thyroidectomy without any complications. Thyroid pathology showed follicular adenoma with a background of hyperplasic benign thyroid parenchyma. She was subsequently discharged home on thyroid hormone replacement.

## Discussion

Thymic lymphoepithelial carcinoma (TLEC) is a rare subtype of thymic carcinoma that is reported in 1.3% to 6% of thymic carcinomas [1].

The association between thymic hyperplasia and Graves disease has been well described in the literature [2]. However, to our knowledge, the coexistence of TLEC and Graves disease has not been previously reported. We describe a rare presentation of a patient with no previous history of thyroid dysfunction who was diagnosed with TLEC and developed thyroid storm postoperatively. We believe that surgery precipitated the thyroid storm in this patient. However, we cannot rule out that the iodine load from contrast administration with prior imaging, could have worsened our patient's undiagnosed Graves disease.

While there is no known association between thymic cancer and Graves disease or thyroid storm, and most cases of thymic cancer arise de novo, there have been reports of secondary thymic squamous cell carcinoma (TSCC) occurring in thymomas or hyperplastic thymic tissue, which can be seen in patients with GD [2, 5]. These secondary TSCC have unique immunohistochemistry characteristics. These include increased expression of epithelial membrane antigen, cytokeratin subtypes, p53 protein, and occasionally CD5 expression changes [5]. TLEC is defined as undifferentiated squamous cell carcinoma [2]; however, whether thymic hyperplasia precedes the development of TLEC tumors is unclear.

It is difficult to ascertain whether our patient had prolonged thymic hyperplasia from pre-existing Graves disease, predisposing her to develop thymic cancer. There are increasing reports of mediastinal tumors associated with Graves disease and thyroid storm [6, 7]. However, there are no clear recommendations to evaluate thyroid function in patients presenting with thymic tumors. In hindsight, appropriate clinical examination and thyroid function testing preoperatively could have possibly prevented thyroid storm in our patient, who developed severe thyroid storm despite appropriate and aggressive treatment with first-line agents.

Multiple factors affected the treatment choices in our patient. Although the patient was started appropriately on methimazole to decrease thyroid hormone synthesis, one could argue that propylthiouracil (PTU) would have added the benefit of inhibiting conversion of T4 to T3. However, methimazole is generally recommended over propylthiouracil due to its safer profile. In addition, the patient was started appropriately on beta-blockers and corticosteroids to ensure inhibition of conversion of T4 to T3. Lugol's iodine solution administration was delayed despite the general recommendations of early administration (at least 1 hour after anti-thyroid drugs). It was mainly due to the concern of a Jod-Basedow phenomenon from the large iodine load patient received from the contrast with her CT scan, which might have worsened her thyrotoxicosis. Although it was less likely that this was a major factor contributing to her worsening clinical status, it was still a possibility, and it may be reasonable to be cautious with any extra iodine load.

Our case also highlights the importance of therapeutic plasma exchange (TPE) in thyroid storm refractory to first-line treatments. TPE involves the removal of a patient's plasma and replacement with allogenic or autologous plasma. TPE was first used in the treatment of thyroid storm in 1970 [8]. The proposed mechanism of action is to directly remove all the mediators of thyroid storm from the plasma, including cytokines, catecholamines, thyroid autoantibodies, and thyroid hormones [9]. Possible complications of TPE include infections, hypocalcemia, hypothermia, hypotension, bleeding diatheses and transfusion reactions.

TPE in thyroid storm is classified as category III by the American Society for Apheresis (ASFA) [10]. ASFA recommends daily TPE sessions with 40 to 50 mL/kg of replacement solution until clinical improvement of thyroid storm. Responses usually vary and tend to be less transient when anti-thyroid drugs have been used prior to TPE initiation [10]. One case series reviewed the use of TPE in 22 patients with thyroid storm and severe refractory thyrotoxicosis; patients received an average of 4 TPE sessions (range, 1-10 sessions), initial thyroid hormone levels decreased by 20% after the first treatment, and by 50% after all sessions [9]. Our patient had a similar response to TPE, with a subsequent decrease in her total T4 levels. Although her total T3 had improved significantly prior to initiation of TPE, her clinical status did not improve until after the TPE, which highlights the role of TPE in removing plasma cytokines and other mediators of thyroid storm.

In conclusion, we present a challenging case of thyroid storm in a patient with a rare subtype of thymic cancer and undiagnosed Graves disease.

## Learning Points

- The case illuminates a novel association of TLEC, and possibly other thymic and mediastinal tumors, with Graves disease.
- Careful history taking and clinical examination prior to surgery are imperative, and evaluation of thyroid function tests should be considered in patients with thymic masses prior to undergoing surgery.
- Use of therapeutic plasma exchange can be critical in the appropriate clinical circumstances, and it is essential to familiarize oneself with its indications and potential risks.

## Contributors

All authors made individual contributions to authorship. K.A. and P.G. were involved in the diagnosis and management of this patient. K.A., M.A., and P.L. were involved in the manuscript submission. All authors reviewed and approved the final draft.

## Data Availability

Data sharing is not applicable to this article as no data sets were generated or analyzed during the present study.

## Abbreviations

ASFA: American Society for Apheresis
CT: computed tomography
TLEC: thymic lymphoepithelial carcinoma
TPE: therapeutic plasma exchange
T3: triiodothyronine
T4: thyroxine
